# Cognitive and Linguistic Predictors of Language Control in Bilingual Children

**DOI:** 10.3389/fpsyg.2020.00968

**Published:** 2020-05-19

**Authors:** Megan C. Gross, Margarita Kaushanskaya

**Affiliations:** ^1^Department of Communication Disorders, University of Massachusetts Amherst, Amherst, MA, United States; ^2^Department of Communication Sciences and Disorders, Waisman Center, University of Wisconsin-Madison, Madison, WI, United States

**Keywords:** bilingualism, children, language switching, language control, cognitive control

## Abstract

In order to communicate effectively with a variety of conversation partners and in a variety of settings, bilingual children must develop language control, the ability to control which language is used for production. Past work has focused on linguistic skills as the limiting factor in children’s ability to control their language choice, while cognitive control has been the focus of adult models of language control. The current study examined the effects of both language ability and cognitive control on language control in 4−6 year old Spanish/English bilingual children with a broad range of language skills, including those with low skills in both languages. To measure language control, children participated in an interactive scripted confederate dialogue paradigm in which they took turns describing picture scenes with video partners who presented themselves as monolingual speakers of English or monolingual speakers of Spanish. The paradigm had two conditions: a single-language context, in which children interacted with only one partner, and a dual-language context, in which children needed to switch between languages to address different partners. The Dimensional Change Card Sort (DCCS) indexed cognitive control. The findings revealed an overall effect of language ability, such that children with lower language skills were more likely to produce words in the language not understood by their conversation partner. There was also an effect of cognitive control on children’s ability to adjust to the dual-language context. Based on these findings, we suggest that a model of language control in children should consider both linguistic and cognitive factors. However, language ability appears to be the main limiting factor, with cognitive control playing a more restricted role in adapting to a dual-language context.

## Introduction

Even as toddlers, bilingual children demonstrate an impressive awareness of their two languages and an emerging ability to control which language is used for production, known as *language control.* Evidence of children’s emerging language control can be most clearly observed through their ability to adjust their language choice to accommodate conversation partners who speak different languages (e.g., Lanza, 1992; Genesee et al., 1995, 1996; Nicoladis and Genesee, 1996; Lanvers, 2001). At this early stage, children show a relative rather than complete adjustment, such as using more English with their English-speaking parent than with their French-speaking parent, but still using some of both languages with both parents. Past work in children has suggested that achieving more complete language control depends on the development of sufficient linguistic skill to express the desired message in the target language (e.g., “bilingual bootstrapping,” Gawlitzek-Maiwald and Tracy, 1996; Lexical Gap Hypothesis, Nicoladis and Secco, 2000). However, limitations in language control are not always explained by linguistic skills (e.g., Nicoladis and Genesee, 1996; Paradis and Nicoladis, 2007; Ribot and Hoff, 2014; Castillo II, 2015). In adult bilinguals, language control has often been associated with cognitive control, with the argument that the ability to control language choice relies on the same cognitive control skills (e.g., inhibition, shifting) that contribute to other aspects of behavior (e.g., Green, 1998; Meuter and Allport, 1999; Green and Abutalebi, 2013). The goal of the current study was to examine an integrated model of language control in children that considers the contributions of both linguistic and cognitive factors.

### Linguistic Predictors of Language Control

When bilingual children produce *cross-language intrusions*, or words in the language not understood by their conversation partner, this lapse in language control has most often been attributed to limited skills in the target language (e.g., Lanza, 1992; Genesee et al., 1995, 1996; Gawlitzek-Maiwald and Tracy, 1996; Nicoladis and Secco, 2000; Lanvers, 2001; Cantone and Mueller, 2005; Ribot and Hoff, 2014). For example, in formulating the Lexical Gap Hypothesis, Nicoladis and Secco (2000) note that very young bilingual children tend to insert words in the non-target language when they do not know the correct word in the target language. With regard to morphosyntax, Gawlitzek-Maiwald and Tracy (1996) suggest a type of “bilingual bootstrapping” through which children use syntactic structures from one language as a placeholder while the analogous syntactic structure in the target language is still developing. Such gaps in lexical and/or syntactic knowledge are a part of typical bilingual acquisition, as bilingual children often show distributed knowledge across their two languages (e.g., Oller et al., 2007; Kohnert, 2010).

Researchers have tended to focus on the role of language-specific knowledge, but several recent studies have considered the role of overall language ability by examining language switching patterns in 5−6 year old bilingual children at risk for Developmental Language Disorder (DLD). While typically developing bilingual children show distributed linguistic knowledge that may result in language-specific gaps, children with language impairment are further challenged by more fundamental difficulties with language learning, processing, and use (e.g., Leonard, 2014; Bishop et al., 2017) that could make it particularly difficult to exercise language control. However, studies have yielded conflicting results as to whether bilingual children at risk for DLD differ from their typically developing peers in their language switching patterns and frequency of their switches into the non-target language.

Some studies (e.g., Gutierrez-Clellen et al., 2009; Greene et al., 2014) have identified no quantitative or qualitative differences in switches out of the target language by Spanish/English bilingual children with low/impaired language compared to typically developing peers during narrative and conversation tasks. Greene et al. (2013) found qualitative but not quantitative differences in language switching during a semantic task. Bilingual children at risk for language impairment were more likely to switch into Spanish than their typically developing peers, who tended to switch into English, the more socially dominant language. Children with low language were also more likely to produce switches that still did not communicate the correct meaning, while children with typical development were more successful in using their switches to improve the accuracy of their responses. Iluz-Cohen and Walters (2012) found both qualitative (directionality and syntactic structure of switches) and quantitative differences in switches out of the target language by Hebrew/English bilingual children with language impairment compared to typically developing peers during narrative tasks. The studies discussed thus far have focused on group comparisons between children with and without language impairment. In a study that only included children with impairment (ages 5−11), Mammolito (2015) found that the tendency to switch into the non-target language during a narrative sample was correlated with overall language ability. Children with more severe impairment (i.e., lower core language skills in both languages) were more likely to switch languages when telling a narrative.

Although both language-specific knowledge and overall language ability have been associated, to at least some extent, with the ability to maintain language control, difficulties with language control cannot fully be explained by language skills (e.g., Nicoladis and Genesee, 1996; Paradis and Nicoladis, 2007; Ribot and Hoff, 2014; Castillo II, 2015). For example, in their study examining children’s ability to adjust their language choice with monolingual strangers, Genesee et al. (1996), Nicoladis and Genesee (1996) found one child who did not make this adjustment, but this child was not the least proficient of the group in the stranger’s language. Genesee et al. (1996) found that, even when children knew both translation equivalents for a given concept, they still sometimes used the English word with a French speaker and the French word with an English speaker. These findings suggest that factors other than linguistic skills should be considered when developing a model of language control in children.

### Cognitive Predictors of Language Control

In addition to being able to express the desired message in the target language, to achieve language control bilinguals also need to monitor the environment for cues, select the appropriate language and inhibit the non-target language, and shift between languages as necessary. These skills (monitoring, inhibiting, shifting) conceptually overlap with *executive functions*, higher-level control processes involved in regulating a variety of behavior (e.g., Miyake et al., 2000; Miyake and Friedman, 2012). Several theoretical models (see Declerck and Philipp, 2015 for a review) suggest a role for domain-general cognitive control skills in language control, including the Inhibitory Control Model (Green, 1998), the Adaptive Control Hypothesis (Green and Abutalebi, 2013), and the Control Processes Model of Code-switching (Green and Wei, 2014). These models include a language schema level (e.g., “speak in English;” “speak in Spanish”) based on the concept of “task sets” from the general task-shifting literature (e.g., Monsell, 2003). While language schemas exert an influence on the language system to help coordinate the processes required for production in the target language, they are believed to be governed *outside* the language system by the same domain-general processes that coordinate any kind of task-shifting.

The relationship between cognitive control and language control has been examined extensively in the adult psycholinguistic literature. Several studies have documented a relationship between cross-language intrusions and measures of inhibition and shifting (e.g., Festman et al., 2010; Gollan et al., 2011, 2014; Festman and Münte, 2012; Prior and Gollan, 2013; Gollan and Goldrick, 2016). For example, bilinguals who more frequently produced words in the wrong language on a cued language switching task were also more likely to perform the wrong task when they were cued to switch between non-linguistic tasks (Prior and Gollan, 2013; Gollan et al., 2014), to make more perseveration errors on the Wisconsin Card Sorting Task (Festman and Münte, 2012), and to take more time on the alternating condition of a Trail-Making task (Gollan and Goldrick, 2016). However, decontextualized picture-naming tasks are far removed from conversational speech and may yield an exaggerated role of cognitive control (e.g., Blanco-Elorrieta and Pylkkänen, 2018).

The role of domain-general cognitive control processes in language control at the conversational level has been formalized in Green and Abutalebi (2013) Adaptive Control Hypothesis. This model of language control still includes a language schema level, as in Green (1998) Inhibitory Control Model, but the way the language schemas are regulated by the domain-general cognitive control system varies depending on the interactional context. When bilinguals operate in single-language contexts, such as using one language at school and another language at home, the language schema for the target language in a given context is activated and the other language schema is inhibited. In contrast, when bilinguals use both of their languages in a dense code-switching context with other bilingual speakers who tend to use both languages within a single sentence, the language task schemas are in a cooperative relationship to allow the integration of elements from both languages. However, when bilinguals use both of their languages in the same context, but with different speakers (i.e., a dual-language context), a competitive relationship between the language schemas exists similar to the single-language context. In addition, there are unique control demands imposed by the need to be prepared to switch the active language schema when addressing speakers of different languages. This dual-language context is the most relevant to the current study, which examined children’s ability to adjust their language choice to accommodate different conversation partners in single-language vs. dual-language contexts. In these specific contexts where there is an expected target language and where language schemas are hypothesized to be in a competitive relationship, instances of language mixing within a sentence (intra-sentential code-switching) would be viewed as cross-language intrusions (use of the language not understood by the current conversation partner). However, it is important to note that intra-sentential code-switching would be entirely appropriate in contexts where the conversation partner also speaks both languages, such as in a dense code-switching context, and the control processes involved may be different. To highlight this distinction, we use the terms *language control* and *cross-language intrusion* rather than *code-switching* to refer to the language behavior under examination in the current study.

The Adaptive Control Hypothesis posits that exercising language control in the dual-language context requires *goal maintenance* to determine the target language (e.g., English), *interference control* to inhibit the non-target language (e.g., Spanish), *detection of salient cues* to determine when a language switch may be necessary (e.g., the arrival of a Spanish-speaking conversation partner), *selective response inhibition* to stop speaking English, *task disengagement* to disengage from the task set for “speak in English,” and *task engagement* to shift to the task set for “speak in Spanish.” Green and Abutalebi (2013) note that there are a variety of multi-model cues to help with these control processes, such as using the voice or face of the addressee to establish the target language (e.g., Woumans et al., 2015; Martin et al., 2016). In addition, in the Interactive Alignment Model of code-switching, Kootstra (2015) and Kootstra et al. (2009) suggest that language activation levels spread from one conversation partner to another so that they align with each other in their language choice. This alignment can be automatic and driven by priming, where listening to a partner speaking one language primes an individual to then use that same language for production. The alignment can also be conscious and strategic based on factors such as prior information about the interlocutor’s language knowledge or preferences. However, Green and Abutalebi (2013) note that there may be other cues in the environment that would be distracting (such as hearing someone else speaking a different language), and thus cognitive control processes are still necessary to coordinate how these bottom-up cues are used.

There are a few studies that have linked cognitive control skills to measures of language control (in terms of cross-language intrusions) in more naturalistic settings. For example, higher self-ratings on questions measuring unintentional language switching in daily life on the Bilingual Switching Questionnaire were associated with poorer inhibitory control, as measured in the lab by a Flanker task (Soveri et al., 2011) or a Stop-signal task (Rodriguez-Fornells et al., 2012). Combining self-report with laboratory measures, Festman (2012) noted that the same bilinguals who demonstrated a relationship between poorer cognitive control (as measured by the Flanker and Wisconsin Card Sorting Task) and increased cross-language intrusions during picture-naming in the lab also provided higher self-report ratings of unintentional switching in daily life. Furthermore, these same individuals produced more cross-language intrusions during a conversation sample in which two interviewers (one who spoke German and one who spoke Russian) alternated about every 5 min when introducing a new topic.

In contrast to the extensive literature on the relationship between cognitive control and language control in adults, very little work has examined the role of cognitive control in the ability of children to exercise language control. It is possible that language control could develop more quickly than cognitive control. For example, children begin to demonstrate the ability to shift from one language to the other based on conversation partner as early as age two (e.g., Nicoladis and Genesee, 1996), while the ability to shift from sorting by color to sorting by shape does not emerge on tasks like the Dimensional Change Card Sort (DCCS) until age 4 or 5 (e.g., Zelazo, 2006). However, early language control involves only relative adjustments in language choice, and it is possible that children’s rapidly developing cognitive control in the preschool years (e.g., Davidson et al., 2006; Huizinga et al., 2006; Garon et al., 2008; Best and Miller, 2010) may play a role, along with their developing linguistic skills, in helping them to achieve more complete language control.

Providing evidence of a relationship between cognitive control and language control in children, our previous work (Gross and Kaushanskaya, 2018) identified cognitive control (as measured by the DCCS) as a significant predictor of cross-language intrusions during picture-naming by 5−7 year old Spanish/English bilingual children. Interestingly, the effect of cognitive control did not interact with the effect of context, indicating that children did not appear to be recruiting cognitive control more when switching between languages in a dual-language context than when using only one language in a single-language context. Although the Adaptive Control Hypothesis suggests that the dual-language context is more taxing for language control, young children who are still developing language control may recruit cognitive control skills to a similar extent to inhibit the non-target language even in a single-language context where no switching is required (see Davidson et al., 2006 for a similar phenomenon in cognitive control tasks). However, a recent study (Kuzyk et al., 2020) found that children’s tendency to switch out of their non-dominant language during a parent-child play sample in a single-language context was not associated with shifting skills as measured by the DCCS but was associated with inhibition skills measured by a Flanker task, which would be more consistent with the control processes posited by the Adaptive Control Hypothesis. The distinction between a picture-naming paradigm (Gross and Kaushanskaya, 2018) and a conversational task (Kuzyk et al., 2020) could be impacting the findings. However, the role of cognitive control in children’s ability to switch between languages in a dual-language context has not been examined in conversational paradigms. Furthermore, these studies of cognitive control and language control were conducted with children with typical language skills and considered the effects of language dominance but not overall language ability.

### Integrating Cognitive and Linguistic Predictors of Language Control

While the Adaptive Control Hypothesis focuses on the role of cognitive control, this model is not necessarily intended as a developmental model and in fact presupposes a high level of proficiency in each language (Green and Abutalebi, 2013). The authors acknowledge that proficiency in each language, as well as variability in cognitive control capacities, may constrain the extent to which individuals are able to adapt their control processes to match the interactional context. Some work in adults suggests that effects of cognitive control on language control are independent of language ability. Festman et al. (2010); Festman (2012) found that bilinguals who produced more cross-language intrusions differed from their fellow participants on measures of cognitive control (e.g., Flanker, Wisconsin Card Sort), but they did not differ on various measures of proficiency in either language (correct responses on verbal fluency tasks, self-ratings of spoken language, quality of language samples). However, these bilinguals were highly proficient in both languages. Even among bilinguals with lower proficiency in their second language, there is some evidence that having better cognitive control skills makes their language control resemble that of more balanced bilinguals (Liu et al., 2014, 2015, 2018).

It is unclear how linguistic and cognitive factors may interact in contributing to language control in earlier stages of development. In children with lower levels of language ability, including those with DLD, limited language ability may constrain language control such that cognitive control does not exert any additional influence. In addition, children with low language may also tend to have lower cognitive control skills. Deficits in inhibition and/or shifting, which are the components of cognitive control most associated with language control, have been demonstrated in both monolingual children with DLD (e.g., Marton, 2008; Spaulding, 2010; Farrant et al., 2012; Henry et al., 2012; Epstein et al., 2014; Kapa and Plante, 2015; Roello et al., 2015; Vissers et al., 2015; Pauls and Archibald, 2016; Sikora et al., 2019) and bilingual children with low language or a diagnosis of DLD (e.g., Iluz-Cohen and Armon-Lotem, 2013; Engel, de Abreu et al., 2014; Sandgren and Holmstrom, 2015; Pauls and Archibald, 2016), although findings have been somewhat mixed with regard to shifting (e.g., Dibbets et al., 2006; Im-Bolter et al., 2006; Laloi, 2015). Therefore, it is possible that low cognitive control could have a negative effect on language control in children with low language, but these effects may be difficult to separate from the effects of limited language ability. In children with higher levels of language ability, based on what has been observed in adults, cognitive control and language ability may have more independent effects on language control. An examination of the contributions of both cognitive control and language ability in children across a broad spectrum of ability is necessary to understand how both cognitive and linguistic factors may contribute to language control.

### Current Study

The current study examined the effects of language ability and cognitive control on language control at the discourse level in young Spanish/English bilinguals (ages 4−6) across a broad range of language ability, including those with low language. We sought to answer the following research questions:

1. What are the contributions of overall language ability and cognitive control to children’s ability to control their language choice across conversation partners and contexts?
2. What are the contributions of overall language ability and cognitive control to children’s ability to adjust to a dual-language context with conversation partners speaking different languages?
3. How do overall language ability and cognitive control interact in their effects on language control?

To examine language control at the discourse level, we designed a computerized scripted confederate dialogue paradigm. The scripted confederate technique has been used in previous studies of linguistic alignment of syntactic choices in monolingual children (Branigan et al., 2005) and in monolingual and bilingual adults (e.g., Branigan et al., 2000; Hartsuiker et al., 2004), including in a study of code-switching behavior (Kootstra et al., 2010). The basic approach is that the participant takes turns identifying pictures described by a partner (the confederate) and describing pictures to the confederate. In the current study, we introduced children to multiple confederates. Some confederates presented themselves as monolingual speakers of English and used English throughout the task, and others presented themselves as monolingual speakers of Spanish and used Spanish throughout the task. Our measure of interest was the extent to which children aligned their language choice to the language spoken by the confederate when they interacted with confederates separately in single-language games and when they interacted with two confederates in a dual-language game. This dual-language game represents the dual-language interactional context that the Adaptive Control Hypothesis (Green and Abutalebi, 2013) describes as recruiting the most cognitive control processes to achieve language control.

Overall language ability was indexed by the Language Index score from the Bilingual English Spanish Assessment (BESA; Peña et al., 2014), which combines children’s best performance across languages on measures of morphosyntax and semantics. Our sample included children with an official diagnosis of language impairment or who may be at risk for language impairment due to low performance in both languages and parent language concerns. However, we chose to analyze language ability as a continuum using the Language Index score rather than as a categorical comparison between children with and without DLD.

We measured cognitive control using a version of the Dimensional Change Card Sort (DCCS) adapted from work by Bialystok and Martin (2004); Zelazo (2006), Zelazo et al. (2013). The DCCS is a complex cognitive control task that requires children to shift from sorting colored shapes by one dimension (e.g., color) to sorting the same stimuli by a different dimension (e.g., shape). This task requires both the ability to *shift* mental sets and the ability to *inhibit* information from the currently irrelevant dimension. In this way, the DCCS taps the same cognitive control skills that may be involved in shifting between languages and inhibiting the non-target language, but in a task that we specifically designed to be as non-linguistic as possible. We use the general term “cognitive control,” rather than specifying specific constructs such as shifting and inhibition, because the goal of the current study was to examine the role of domain-general cognitive control and not necessarily to pinpoint the specific processes involved. In addition, the relationship between shifting and inhibition may be complex, especially in young children (e.g., Garon et al., 2008; Best and Miller, 2010).

Based on past work on language control in children, we expected that the ability to exercise language control during our task would be predicted by overall language ability, such that children with stronger language skills overall would be more successful in controlling their language choice. It was difficult to predict the role of cognitive control given the paucity of research on cognitive control and language control in children. Based on our past work at the single word level (Gross and Kaushanskaya, 2018), we would expect cognitive control to have an overall effect on language control. If the Adaptive Control Hypothesis can be applied to children, then we would expect an interaction with context such that cognitive control would be especially associated with language control in a dual-language context. Finally, we expected an interaction between the effects of language ability and cognitive control such that cognitive control would make a more independent contribution to language control in children with higher levels of language ability.

## Materials and Methods

### Participants

The current study included sixty-two Spanish-English bilingual children (25 boys), ages 4; 0−6; 11 (*M*_age_ = 5.35 years; *SD* = 0.93). All children acquired Spanish from birth and were exposed to English either simultaneously with Spanish within their first year (*n* = 42) or sequentially after 18 months (*n* = 20). All children passed a pure-tone hearing screening at 20 dB at 1000, 2000, and 4000 Hz in each ear and had non-verbal intelligence scores within normal limits. Table 1 presents participant characteristics. These children had a broad range of language ability (*M* = 102.23, *SD* = 13.14, *range* = 71−126), as measured by the Language Index score from the *Bilingual English-Spanish Assessment* (BESA; Peña et al., 2014). Thirteen children were flagged as having low language skills based on receiving a morphosyntax score in their better language that was at or below the empirically derived cut-off in the BESA manual for their age group. Eight children had an existing diagnosis of language impairment or history of language services, and 32 children had parent language concerns. A total of 15 children met at least 2 out of these 3 criteria, which was our operational definition for DLD. However, in the current study, language ability was measured on a continuum using the BESA Language Index Score as a measure of overall language ability, rather than creating discrete diagnostic groups.

**TABLE 1:** Language background characteristics for participants (*n* = 62).

Characteristic	Mean (*SD*)
Age of First English Exposure (months)	12.15 (15.37) [Range: 0−48]
Current Spanish Input/Output (% of waking hours)^*a*^	54% (16) [Range: 24−84]
Language of Instruction at School/Daycare	Spanish: 4, English: 27, Both: 28
	No school/daycare: 3
Maternal Education (1−6 scale)^b^	3.13 (1.76) [Range: 1−6]
Non-verbal Intelligence Std. Score (Leiter-3)	104.11 (7.51) [Range: 87−123]
BESA Spanish Morphosyntax Std. Score	87.24 (17.69) [Range: 55−123]
BESA Spanish Semantics Std. Score	104.23 (13.22) [Range: 73−130]
BESA English Morphosyntax Std. Score	93.95 (17.61) [Range: 62−118]
BESA English Semantics Std. Score	100.48 (14.20) [Range: 65−123]
BESA Language Index^c^	102.23 (13.14) [Range: 71−126]

*^*a*^From the Bilingual Input Output Survey (BIOS) in the Bilingual English Spanish Assessment (BESA), completed during the parent interview. Spanish input/output represents an average of the proportion of waking hours during which a child hears Spanish and speaks Spanish. Time periods when both Spanish and English are heard/used are treated as 50% Spanish in the calculation of Spanish input/out, regardless of the actual language breakdown during this time. ^*b*^Scale: 1 = < HS, 2 = HS/GED, 3 = some college/2-year degree, 4 = BA, 5 = MA, 6 = Doctorate. ^*c*^The Language Index represents overall language ability and is derived by combining the child’s highest Morphosyntax score (English or Spanish) and highest Semantics score (English or Spanish). For a child with mixed dominance, the Language Index could combine, for example, morphosyntax in English and semantics in Spanish.*

Exclusionary criteria included hearing impairment, neurological impairment, genetic syndromes, psychological/behavioral disorders, other developmental disabilities, current exposure to a language other than English or Spanish (>5% of waking hours), or significant past exposure (e.g., daycare provider spoke a third language to the child). ADHD and speech sound disorders were not considered to be exclusionary criteria. As these conditions often co-occur with language impairment, variation in attention and speech sound production was permitted throughout the range of language ability. Sixteen additional children completed the experimental tasks but were excluded from the final analysis for the current study due to failing the hearing screening (*n* = 3), suspected neurological impairment (*n* = 1), growing up abroad with more diverse language exposure than the rest of the sample (*n* = 3), acquiring Spanish after birth and/or not having a caregiver who speaks Spanish (*n* = 4), demonstrating extremely limited English or Spanish expressive skills compared to the rest of the sample in a vocabulary post-test associated with the main experimental task (*n* = 3), or producing null responses or “I don’t know” on all trials within a condition (*n* = 2).

### General Procedure

The study was completed over three or four 1−1.5 h individual sessions in a laboratory setting at the Waisman Center. The study was approved by the Institutional Review Board (IRB) of the University of Wisconsin-Madison. Parents provided written consent and children provided verbal assent prior to beginning the study. The three versions of the scripted confederate dialogue task (single-language English, single-language Spanish, dual-language) were each administered at the beginning of a session. Sessions were scheduled at least 1 week apart. To avoid confounding the effects of dual-language context with order effects, the dual-language game was presented in the first session for approximately half of the children (*n* = 28) and in the third session for the rest (*n* = 34). The order of the single-language games was determined based on the child’s preferred language (as expressed by the parent or the child: 32 English first, 30 Spanish first). The standardized assessments of vocabulary, language ability, and non-verbal intelligence were distributed across sessions. The cognitive control measure, a computerized Dimensional Change Card Sort, could be administered in any session, as long as it occurred after the children had completed the dual-language version of the scripted confederate dialogue task.

Parents (46 mothers, 16 fathers) were interviewed in their preferred language about their child’s development, medical and educational history, language history, and current language use and exposure. Parents also completed the Bilingual Input Output Survey (BIOS) as a measure of current language exposure and the Inventory to Assess Language Knowledge (ITALK) as a measure of parent language concerns (Peña et al., 2014). The BIOS provides an average Spanish input/output percentage by asking parents to indicate, for each hour that their child is awake on a typical weekday and weekend, what language the child hears (English, Spanish, or both) and what language the child speaks. The formula treats exposure to “both” as 50% Spanish and 50% English, but parents sometimes indicated that periods of dual-language exposure were not necessarily balanced. In addition, maternal level of education (as a proxy for socioeconomic status) was measured on a Likert scale (1 = less than high school, 2 = high school or GED, 3 = 2-year degree or some college; 4 = Bachelor’s degree, 5 = Master’s degree, 6 = Doctoral degree).

### Standardized Assessments

The *Leiter International Performance Scale* (Leiter-3; Roid et al., 2013) was administered to ensure that all participants had non-verbal intelligence within normal limits (i.e., >85). To measure language ability, children completed the English and Spanish morphosyntax and semantics subtests from the *Bilingual English-Spanish Assessment* (BESA; Peña et al., 2014). The higher morphosyntax score (whether in English or Spanish) was combined with the higher semantics score to obtain a Language Index score. For a child with mixed dominance, the Language Index could reflect, for example, a combination of morphosyntax skills in English and semantics skills in Spanish. Children are also permitted to code-switch during the assessment, such that they can receive credit for English responses on the Spanish semantics subtest (and vice versa), as long as the answer demonstrates understanding of the question. The Language Index is intended to provide a global measure of underlying language ability that is not specific to a given language or domain (Peña et al., 2014). In the current study, the Language Index was used as a continuous variable to index overall language ability. Children also completed the *Expressive One-Word Picture Vocabulary Test – 4: Spanish-Bilingual Edition* (EOWPVT-4 SBE, Martin, 2013), which gives them the opportunity to respond in English or Spanish for any given item. The final score was not used in analyses in the current study, as it was highly correlated with the BESA Language Index score (*r* = 0.82), and we were interested in the effects of broad language ability rather than lexical skills in particular. However, the proportion of items named in each language during the EOWPVT helped to establish language dominance.

### Scripted Confederate Dialogue Task

Children participated in a computerized scripted confederate dialogue task to assess their language control abilities. Additional details about the development and norming of the paradigm and stimuli are presented in the Supplementary Material.

### Procedure

Children were told that they would play a game with someone in another room, and a video of the confederate was presented to the child on a computer screen. All confederates presented themselves to the children as monolingual speakers of English or Spanish (e.g., “My name is Ashley and I only speak English;” “Me llamo Maria y sólo hablo español”). The confederate videos were pre-recorded to preserve experimental control, but feedback contingencies were programed into the experiment so that the interaction would seem as natural as possible. Children’s behavior (e.g., waving or making unsolicited comments to the partner) suggested that they believed the interaction was occurring in real time. Children played three games in three separate sessions, with at least 1 week between sessions: (1) *single-language* with an English-speaking partner, (2) *single-language* with a Spanish-speaking partner, and (3) *dual-language* with turns alternating pseudo-randomly between a new Spanish-speaking partner and a new English-speaking partner. The order of the single-language games was based on the child’s language preference, and the order of the dual-language game (first or last) was counterbalanced across participants.

**FIGURE 1:**
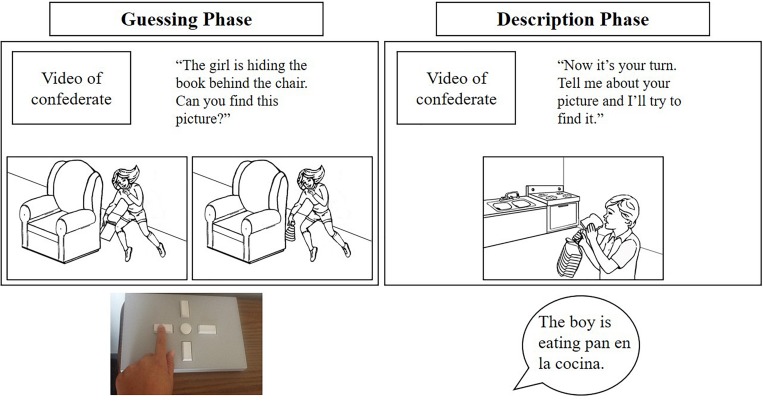
Experimental set-up for one trial in the English version of the scripted confederate dialogue task. The boxes show what the child saw on the computer screen. Text is included for demonstration purposes only; the child only saw the picture scenes and a video of the confederate. Below the boxes are sample responses from the child, pushing the correct button in the guessing phase and producing a cross-language intrusion in the description phase.

The task was presented using E-Prime 2.0 (build 2.0.10.242, Psychology Software Tools, 2012) on a desktop computer with a 23-inch monitor and a resolution of 1920 × 1080. Each game included 20 trials composed of a guessing phase and a description phase. During the guessing phase, the child saw two pictures and the confederate produced a sentence describing one of them (e.g., “The boy is watching the airplane in the sky. Can you find this picture?”). The child had 20 s to push a button on a serial response box to indicate which picture the confederate was describing. During the description phase, the child saw one picture and was instructed to describe it to the confederate (e.g., “Now it’s your turn. Tell me about your picture and I’ll try to find it.”). If the child produced a description within the 30-s window, the confederate acknowledged the response (e.g., “Thanks! I’ll try this one”) and pushed a button on her own button box. If the child did not produce a description or indicated “I don’t know,” the confederate reminded the child to try to say something about the pictures, and the experiment proceeded to the next trial. Figure 1 shows a schematic of the guessing and description phases of a trial. When possible, the experimenter noted a rough transcription and the language(s) used by the child on each trial. Audio and/or video recordings (depending on parent permission) were made for later coding. To provide motivation, children were told they would earn a star each time they found the confederate’s picture and each time the confederate found their picture. Every five trials children received a break and saw how many stars they had earned (randomly generated to show progress, but not contingent on actual accuracy), and at the end of the game they got to pick one sticker for every ten stars earned.

The experimenter provided a brief overview of the game in the language of the task for the single-language games (unless the child specifically requested otherwise) and in the child’s preferred language for the dual-language game. Then the video confederate introduced herself and demonstrated how to play the game through two practice trials. In the dual-language version, the confederate who spoke the child’s preferred language introduced the game and presented the first practice trial, and the confederate who spoke the other language presented the second practice trial. Beyond the presentation of the confederates as monolingual speakers, children were never explicitly told to speak a certain language. If they asked which language to use, they were encouraged to speak so that their partner would understand. The experimenter spoke as little as possible during the task, prompting the child when necessary to maintain on-task behavior. To create a consistent language environment, the experimenter generally used the same language as the current confederate, but the children knew the experimenter was bilingual and sometimes addressed the experimenter in a different language from the confederate. The task was administered by the first author, a highly proficient non-native speaker of Spanish, so that any influence of the experimenter’s linguistic background on the language choice of participants would be consistent.^1^

#### Materials

The task included 20 pairs of picture scenes that differed in one element (subject, object, or location) for the guessing phase, as well as 20 scenes for the child to describe back to the confederate in the description phase. The scenes were created in Adobe Photoshop CC 2015 and consisted of object and action images from the International Picture Naming Database (Snodgrass and Vanderwart, 1980; Szekely et al., 2004; Center for Research in Language, 2014) and similarly styled clipart or manual drawings. The sentences produced by the confederate were 8−11 words long and followed the structure NP VP NP PP (e.g., The girl is hiding the book behind the chair). The description scenes were constructed with animate subjects performing an action on an object in a location (or for a recipient) so that they could be naturally described with this structure as well. The description scenes and guessing scenes were designed to have minimal overlap to reduce lexical priming effects. The scene elements (subject, verb, object, location) were selected to have English and Spanish labels that were non-cognates, early-acquired (CLEX database for acquisition norms from the American English and Mexican Spanish versions of the MacArthur Communicative Development Inventories, Dale and Fenson, 1996; Jackson-Maldonado et al., 2003; Center for Child Language, 2013) and high-frequency [at least 10 tokens per million in the Corpus of Contemporary American English (Davies, 2008) and the Corpus del Español (Davies, 2002)]. The subjects, verbs, objects and location elements included in the description scenes are listed in Appendix A. At the end of the final session, children completed a vocabulary post-test to assess their knowledge of the English and Spanish words for these scene elements.

Each condition (English, Spanish, and Dual-Language) included the same set of stimuli but in a different pseudorandomized order (Research Randomizer, Urbaniak and Plous, 2013) in which none of the elements (subject, verb, object, location) of a description scene repeated in consecutive trials. The guessing scene pairs and description scenes were yoked such that a given description scene always followed the same guessing scene pair in each condition. The yoked pairs were carefully selected to ensure no lexical or semantic overlap between the sentence that the child heard and the picture that the child needed to describe. For the dual-language condition, half of the trials were presented by the English-speaking confederate and half were presented by the Spanish-speaking confederate. The sequence of English and Spanish trials was pseudo-randomized to ensure no more than four consecutive trials in a single language and to ensure that half of the trials required a switch in languages from the previous trial. Two versions of the dual-language block were created such that trials presented by the English-speaking confederate in version A were presented by the Spanish-speaking confederate in version B, and vice versa. Thus, a given item occurred in the dual-language condition in only one language for a single participant, but it was presented in both languages across participants (31 children received each version). Appendix B shows the yoked pairs of guessing and description scenes in the pseudorandom sequence designed for the dual-language block, version A.

Four adult females (two functionally monolingual English speakers and two functionally monolingual Spanish speakers from Mexico) recorded the confederate videos. Children were assigned (based on the sequence of their participant ID number) to a combination of English, Spanish and dual-language versions where the confederates in the dual-language block were distinct from the confederates in the single-language blocks (e.g., English S1; Spanish S2; Dual-Language with English S2 and Spanish S1).

#### Transcription and Coding

Each picture description provided by the child was transcribed using the Systematic Analysis of Language Transcripts (SALT; Miller and Iglesias, 2017) by three bilingual research assistants (one native speaker of Spanish and two highly proficient non-native speakers of Spanish). All words produced by the child in the language not spoken by the current confederate were coded as [CS]. Blends containing features of both languages (e.g., queso + cheese = /kiz/) were coded as [CS]. Preliminary transcriptions had been completed by the first author while the child was performing the task or during a review of the audio/video by a research assistant while broadly coding the language of the child’s response. These preliminary transcripts served as an initial guide for the detailed transcription in SALT, but the transcriber reviewed each audio or video file to account for all words spoken by the child. False starts, reformulations, repetitions, and side comments clearly addressed to the experimenter or as self-talk were not counted as part of the picture description for the confederate.

During training on transcripts from 4 to 5 different children, each transcriber demonstrated inter-rater agreement (compared to another transcriber or the first author) of 98% for total words produced by the child, at least 90% for total words in the non-target language, and 100% for total utterances containing at least one word in the non-target language. A fourth bilingual research assistant assisted with checking procedures. All transcripts were double-checked for accuracy of transcription conventions and completeness, referring back to the audio/video as needed to clarify what the child had said. Disagreements about whether a word was produced in the non-target language were resolved by consensus. Finally, word lists were generated across all transcripts to identify words in the non-target language that had not been marked with [CS].

In the current study, language control was measured in terms of cross-language intrusions, which were defined at the utterance level as picture descriptions containing at least one word in the non-target language. Thus, picture descriptions with at least one word marked as [CS] received a code of “1” and picture descriptions entirely in the target language received a code of “0.” Exploratory analyses revealed that a more graded coding system of proportion of [CS] words out of total words yielded similar information to this binary coding system.

Trials were excluded from the analysis if the child did not provide a response (*n* = 11 trials), indicated that he or she did not know what to say (*n* = 44 trials), provided a response with words that were too unintelligible to identify the language (*n* = 18 trials), or provided an entirely unrelated response that was not an attempt to describe the picture (*n* = 12 trials). An additional three trials were excluded due to technical failure or because the child needed to leave the room before one of the scheduled breaks. Overall, these exclusions resulted in the loss of 2.37% of the total trials, and the analyses included a total of 3632 trials across 62 children and 20 different description scenes.

For the analyses, the language of each trial was re-coded from English vs. Spanish to “dominant” vs. “non-dominant” based on each individual child’s dominant language. This coding convention is commonly used in language switching studies when a sample contains participants with different dominance profiles (e.g., Prior and Gollan, 2011; Weissberger et al., 2012). Dominance is a complex construct and is often mixed depending on the area of language under examination (e.g., Bedore et al., 2012). In the current study, a broad measure of dominance was determined by examining seven indicators: current exposure (Spanish input/output as calculated from the BIOS), parent-reported dominance, child preference (the language in which children preferred to start the study), expressive vocabulary (the language used on the majority of items during the Expressive One Word Picture Vocabulary Test, which allows children to respond in either language), expressive morphosyntax (the higher Morphosyntax score on the BESA), receptive language (the higher receptive Semantics score on the BESA), and broad language (the higher Language Index score on the BESA, calculated within each language separately). Children were classified as English-dominant (*n* = 36) if the majority of indicators (excluding ties) pointed to English and as Spanish-dominant (*n* = 26) if the majority pointed to Spanish.

### Dimensional Change Card Sort (DCCS)

As a measure of cognitive control, children completed a version of the Dimensional Change Card Sort (DCCS) that integrated components of the color-shape game used by Bialystok and Martin (2004) and the DCCS task created for the NIH toolbox (Zelazo et al., 2013). This version of the DCCS was initially designed for a project examining language and executive function in older children (ages 8−11) with typical language, specific language impairment, and autism spectrum disorder (e.g., Kaushanskaya et al., 2017), but versions of the DCCS have often been used with 4−6 year old children (e.g., Frye et al., 1995; Bialystok and Martin, 2004; Zelazo, 2006; Zelazo et al., 2013). Our version was designed to reduce linguistic demands by using simple red circles and blue squares as stimuli, pairing initial verbal instructions (in the child’s preferred language) with photographs that illustrated what to do, and using non-linguistic sorting cues (a row of amorphous color patches for sorting by color and a row of gray circles and squares for shorting by shape). The cues remained throughout the trial to reduce working memory demands.

The DCCS was presented using E-Prime 2.0 on a desktop computer with a 23-inch monitor. For each trial, the sorting cue appeared at the top of the screen, and, after 500 ms, the stimulus (a red circle or blue square) appeared in the center of the screen while the cue remained at the top. Throughout the task, gray response buckets marked with a red square and a blue circle were present at the left and right bottom corners of the screen. Children were instructed to sort the stimulus into one of the buckets by pressing the left or right button on a serial response box. Following the child’s response, or at the end of the 10-s response window, the next trial began after an inter-trial interval of 800 ms.

The task included three phases: pre-switch, post-switch, and mixed. During the *pre-switch* phase, the children were introduced to the “color game” by showing them how to sort the blue square into the bucket marked with the blue circle and the red circle into the bucket marked with the red square by pushing the corresponding buttons. To ensure that children understood the basic idea of pushing a button to sort the stimuli, they completed four practice trials with feedback, and the instructions and practice were repeated if children made more than one mistake. Then the child completed the 5 pre-switch trials with no feedback. In the *post-switch* phase, children had to shift from sorting by color to sorting the same stimuli by shape. To respond correctly, children had to *shift* mental sets to the new dimension and *inhibit* their attention to color and the prepotent response to sort by color. Children were introduced to this new “shape game” with an example of how to sort each stimulus, but they completed the 5 post-switch trials with no practice to avoid diluting the effect of the shift in sorting rules. All children advanced from the pre-switch to the post-switch phase, regardless of performance on the pre-switch phase. Children also completed a *mixed* phase (30 trials) in which the sorting rule switched periodically. However, this phase was too difficult for children in the current study and was not included in the analysis.

Performance on the post-switch phase was the primary outcome measure and was scored on a pass/fail basis. In young children, prior work has suggested that accuracy may better index performance than reaction time (e.g., Diamond and Kirkham, 2005; Davidson et al., 2006). Use of a pass/fail metric is consistent with other studies of the DCCS in young children (e.g., Rennie et al., 2004; Diamond et al., 2005; Zelazo, 2006) and with the distribution of responses in the current study. Children who responded correctly on 4/5 trials (*n* = 40) were considered to pass, and all other children (*n* = 22) were considered to fail. In keeping with developmental expectations, children who passed the DCCS were significantly older (*M* = 5.52, *SD* = 0.90) than children who failed (*M* = 5.03, *SD* = 0.94), *t*(65) = 2.01, *p* = 0.049. They did not differ on other variables, including maternal education, non-verbal IQ, language ability, English age of acquisition, or current language exposure (all *p*s > 0.30).

### Analyses

To address the research questions about predictors of language control, mixed effects logistic regression models were constructed in which the outcome variable was the odds of a child producing a cross-language intrusion (coded as “1” vs. “0”) when describing a picture to a conversation partner in a monolingual context. The initial base model examined the effects of task-level variables (i.e., whether the conversation partner spoke the child’s dominant or non-dominant language; whether the interaction took place in a single-language vs. dual-language context) and child-level covariates (i.e., age, maternal education, current Spanish input/output). Maternal education was indexed by the highest level of education completed by the child’s mother, on a 1−6 Likert scale. Next, we tested for any significant effects of counterbalanced manipulations, including the version of the dual-language condition (A vs. B) and the order in which the dual-language condition was administered (in the first session vs. the last session).

To address the first research question about the overall effects of language ability and cognitive control on language control, main effects of language ability (operationalized as the Language Index score from the BESA) and cognitive control (operationalized as a dichotomous pass/fail measure from the DCCS post-switch phase) were added to the model. To address the second research question about whether language ability and/or cognitive control moderated children’s ability to adapt to the dual-language context, the interaction between context and language ability and between context and cognitive control were each added to the model. To address the third research question about interrelated effects of cognitive control and language ability, the three-way interaction among context, language ability, and cognitive control was added to the model.

All models were evaluated using the glmer() function from the lme4 package (Bates et al., 2015; version 1.1-21) in R version 3.6.1 (R Core Team, 2019). Models were initially constructed with a maximal random effects structure (e.g., Barr et al., 2013), including random intercepts for participants and items, and random by-participant and by-item slopes for context and partner language. However, to resolve singularity warnings, the random effect with the smallest variance (by-items random slope for context) was removed. For fixed effects, the significance of a given predictor was established through a likelihood ratio chi-square test comparing the full model to a restricted model with the focal predictor removed (Bolker, 2014, 2018; Social Science and Computing Cooperative, 2016). For each predictor, model tables report the unstandardized coefficient estimate (log-odds scale), the standard error, and the results of the likelihood ratio chi-square test evaluating the significance of the predictor. Dichotomous predictors were sum coded as −0.5 and 0.5. Continuous variables were centered and scaled (i.e., standardized) to promote model convergence.

## Results

### Base Model

Descriptive data with the mean proportion of cross-language intrusions in each condition are reported in Table 2. The base model examined the task manipulations of context (single-language vs. dual-language) and partner language (child’s dominant vs. non-dominant language) and potential covariates (age, maternal education, Spanish input/output). There was a robust effect of partner language [χ^2^(1) = 21.24, *p* < 0.001, *b* = 6.01, *SE* = 1.39], such that children were more likely to produce cross-language intrusions when interacting with a partner who spoke their non-dominant language. The effect of context was not significant [χ^2^(1) = 1.45, *p* = 0.23, *b* = 0.53, *SE* = 0.43] and neither was the interaction between partner language and context [χ^2^(1) = 1.89, *p* = 0.17, *b* = 0.85, *SE* = 0.62]. Age had a significant effect on language control [χ^2^(1) = 10.41, *p* = 0.001, *b* = −1.48, *SE* = 0.47], with older children less likely to produce cross-language intrusions. Children with higher maternal education levels were significantly less likely to produce cross-language intrusions, χ^2^(1) = 10.26, *p* = 0.001, *b* = −1.39, *SE* = 0.44. Spanish input/output did not have a significant effect on language control [χ^2^(1) = 0.75, *p* = 0.39, *b* = −0.42, *SE* = 0.50]. When Spanish input/output and the interaction between partner language and context were removed from the model, the main effect of context increased (*b* = 0.79, *SE* = 0.39), but did not reach significance when assessed by a likelihood ratio test [χ^2^(1) = 3.70, *p* = 0.054].

**TABLE 2:** Mean (*SD*) for the proportion of cross-language intrusions in each condition, averaged over participants.

Context	Partner language	
	Child’s dominant language	Child’s non-dominant language
Single-Language	0.06 (0.18)	0.35 (0.42)
Dual-Language	0.07 (0.18)	0.43 (0.43)

In a model evaluating counterbalanced manipulations, adding version (A vs. B) and its interaction with context did not improve the model [χ^2^(2) = 0.056, *p* = 0.97]. Similarly, adding the order in which children completed the dual-language condition (first vs. last) and its interaction with context did not improve the model [χ^2^(2) = 1.47, *p* = 0.48]. Therefore, version and order were not included in any subsequent models.

In summary, age and maternal education were identified as covariates to include in subsequent models. The predominant effect identified in the base model was the effect of partner language, revealing the large impact of language dominance. By including this effect in subsequent models, we examine whether there are other significant predictors of language control, over and above the tendency to produce cross-language intrusions when interacting in the non-dominant language. While the effect of context did not reach significance in the base model, subsequent models explored potential moderators of the effect of context.

### Overall Effects of Language Ability and Cognitive Control on Language Control

Controlling for the effects of age and maternal education, language ability had a significant main effect on language control [χ^2^(1) = 6.57, *p* = 0.01, *b* = −1.33, *SE* = 0.56]. For a decrease of one standard deviation below the average BESA Language Index score, the odds of producing a cross-language intrusion are predicted to increase by a factor of 3.78 (95% CI: 1.28 – 11.25). When language ability was added to the model, adding maternal education no longer significantly improved the model [χ^2^(1) = 1.84, *p* = 0.17]. The Language Index score was correlated with maternal education (*r* = 0.54, *p* < 0.001). However, this relationship was not so strong as to raise concerns of multicollinearity. Both predictors were retained in order to evaluate the effects of language ability after accounting for the effects of maternal education. Furthermore, the effect of language ability persisted even when children with below-average language skills or with a history of language therapy were removed [χ^2^(1) = 7.26, *p* = 0.007, *b* = −1.52, *SE* = 0.58], suggesting that language ability has an effect on language control throughout the continuum of ability and not just for children with language difficulties. Adding a main effect of cognitive control did not improve the model [χ^2^(1) = 0.33, *p* = 0.57]. Therefore, language ability appears to have an overall effect on language control, while cognitive control does not.

### Moderating Effects of Language Ability and Cognitive Control

**FIGURE 2:**
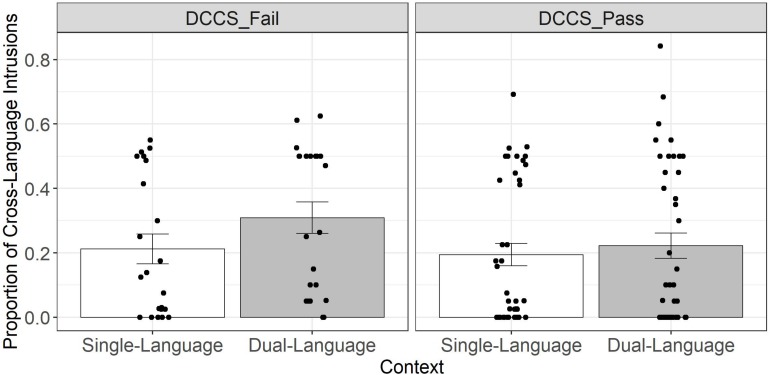
Cross-language intrusions as a function of context (single-language vs. dual-language) for children who failed the DCCS post-switch condition (*n* = 22) and children who passed the DCCS post-switch condition (*n* = 40). Plots present raw data (aggregated by participant) and were created in R using the ggplot2 package (Wickham, 2009; version 3.2.1). Bars represent condition means (averaged over participants) and error bars reflect one standard error. Data points represent individual participants (*n* = 62).

Adding the interaction between language ability and context to a model containing only main effects did not significantly improve the model [χ^2^(1) = 1.67, *p* = 0.20]. However, adding the interaction between cognitive control and context did result in a significant improvement [χ^2^(1) = 4.03, *p* = 0.045, *b* = 1.57, *SE* = 0.80]. As shown in Figure 2, children with lower cognitive control skills who failed the DCCS showed a significantly larger effect of context than children who passed the DCCS. This pattern was confirmed by re-running the model with cognitive control dummy-coded instead of sum-coded and changing the reference category. With failing the DCCS as the reference category, there was a robust effect of context (*b* = 1.72, *SE* = 0.62), such that the odds of producing a cross-language intrusion increased by a factor of 5.6 (95% CI: 1.65 – 18.8) in the dual-language context compared to the single-language context. In contrast, when passing the DCCS was the reference category, the effect of context was minimal (*b* = 0.15, *SE* = 0.50). Table 3 shows the final optimal model containing a main effect of language ability and a moderating effect of cognitive control on the effect of context.

**TABLE 3:** Mixed-effects logistic regression model of linguistic and cognitive predictors of the odds of producing a cross-language intrusion.

Variable	Estimate (log-odds)	*SE*	χ*^2^*	*p*-Value
Intercept	− 4.65	0.75		
Age (standardized)	− 1.52	0.48	10.33	0.001
Maternal Education (standardized)	− 0.71	0.50	1.92	0.166
Partner Language (dom[−0.5] vs. non-dom[0.5])	6.60	1.61	22.99	< 0.001
Context (single[−0.5] vs. dual[0.5])	0.93	0.40	4.94	0.026
Language Index (standardized)	− 1.34	0.55	4.00	0.045
DCCS (pass[−0.5] vs. fail[0.5])^a^	− 0.20	0.93	0.05	0.829
DCCS X Context	1.57	0.80	4.03	0.045

*^a^Dimensional Change Card Sort post-switch phase.*

### Interrelated Effects of Language Ability and Cognitive Control

Adding a two-way interaction between language ability and cognitive control did not improve the model [χ^2^(1) = 0.04, *p* = 0.84], suggesting that the overall effect of language ability is not moderated by cognitive control. The 3-way interaction among language ability, cognitive control, and context, also did not improve the model [χ^2^(1) = 2.29, *p* = 0.13, *b* = 1.20, *SE* = 0.80]. Thus, the interaction between context and cognitive control was not further moderated by language ability.

## Discussion

The goal of the current study was to test a framework for understanding children’s language control that included both linguistic and cognitive factors. In terms of linguistic factors, there was a robust effect of language dominance, such that children were far more likely to produce cross-language intrusions when interacting with a partner who spoke their non-dominant language. In addition, over and above the effects of language dominance, we were particularly interested in the role of overall language ability in a broad sample ranging from children with impaired language to those with superior language skills. We found that overall language ability had a significant effect on language control, such that children with better language skills were less likely to produce cross-language intrusions during the scripted confederate dialogue task. This effect did not interact with context, indicating that language ability predicted language control overall, but it did not play a greater role in language control in the dual-language context than the single-language context. With regard to cognitive control, we observed the opposite pattern. Cognitive control did not have an overall effect on language control, but it did interact with context. Children with lower cognitive control showed a larger increase in cross-language intrusions in the dual-language context relative to the single-language context. Furthermore, this moderating effect of cognitive control did not depend on children’s level of language ability.

### The Effect of Language Ability on Language Control

The finding that overall language ability is a continuous predictor of cross-language intrusions contributes to the current literature about linguistic predictors of language control in bilingual children. Past work in children with typical language development has focused on language-specific skills as constraining children’s ability to adjust their language choice to accommodate the current conversation partner or language context (e.g., Lanza, 1992; Genesee et al., 1995, 1996; Gawlitzek-Maiwald and Tracy, 1996; Nicoladis and Secco, 2000; Lanvers, 2001; Cantone and Mueller, 2005; Ribot and Hoff, 2014). Contributions of overall language ability to language control have been examined mostly in children with language impairment (e.g., Gutierrez-Clellen et al., 2009; Iluz-Cohen and Walters, 2012; Greene et al., 2013, 2014; Mammolito, 2015). The current study demonstrated a relationship between overall language ability and language control in bilingual children across a broad spectrum of ability ranging from impaired to above-average.

Why might overall language ability affect language control? One possibility is that language control is part of the overall integrity of the bilingual language system and is a component of language use that develops as children gain competence as communicators. If the goal of conversation is to achieve mutual understanding through *interactive alignment* (e.g., Garrod and Pickering, 2004; Kootstra et al., 2009; Kootstra, 2015), then this goal will be most successfully achieved if children use the language that their listener will understand best. Otherwise, they will experience an “interaction cost,” or a disruption to the conversation, which Green and Abutalebi 2013, p. 521) describe as the motivation for exercising language control. Sensitivity to this interaction cost may be related to overall language ability. Such a relationship would be in line with the observation by both Iluz-Cohen and Walters (2012); Greene et al. (2013) that children with language impairment may be less sensitive to sociolinguistic context.

It is also possible that the effect of overall language ability in the current study was driven by language-specific knowledge. Children with lower overall language ability may have more lexical/syntactic gaps in each language, while children with higher overall language ability may have fewer lexical/syntactic gaps, even in their weaker language. To examine whether overall language ability plays an independent role in language control, over and above the effects of language-specific knowledge, further analysis controlling for lexical gaps is necessary. If two children with similar lexical gaps in their less dominant language, but different levels of overall language ability, still show differences in language control, then this would lend support to a role for overall language ability in supporting language control. As evidence that lexical gaps may be dissociated from overall communicative competence, Barbosa et al. (2017) found that typically developing French/English bilingual children knew fewer target words needed to retell a narrative in English than their monolingual peers, but they used compensatory strategies (e.g., using a superordinate category word or circumlocution) and still included as many or more key concepts in their stories.

Although language ability had an overall effect on language control in the current study, it did not affect children’s ability to adapt to the dual-language context, as the interaction between language ability and context was not significant. These findings would suggest that, regardless of children’s level of language ability, they did not have more difficulty with language control in a dual-language context than in a single language context. Notably, the main effect of context was not significant in the present study. Even if the main effect of context could be considered borderline [*b* = 0.79, *SE* = 0.39; χ^2^(1) = 3.70, *p* = 0.054], it is still much less robust than has been observed in our previous work at the single-word level in a picture-naming paradigm [χ^2^(1) = 23.95, *p* < 0.001, *b* = −1.55, *SE* = 0.36] with children from a similar population (Gross and Kaushanskaya, 2018).

The scripted confederate dialogue paradigm differs from a decontextualized picture-naming task in a variety of ways that could have facilitated language control in a dual-language context for children with a spectrum of language abilities. A picture naming paradigm provides only a brief auditory cue (“Say” vs. “Diga”) to indicate the target language. In the scripted confederate paradigm, the appearance of a new speaker may help to signal an upcoming change, and cues from her appearance and prior knowledge of what language she speaks may help children to anticipate what the target language should be (e.g., Woumans et al., 2015; Martin et al., 2016). In addition, children have the opportunity to listen to their partner describe a picture in the target language before they have to produce anything in that language themselves. In the interactive alignment model of code-switching, Kootstra et al. (2009) and Kootstra (2015) suggest that language activation can spread from one conversation partner to another through priming to facilitate alignment of language choice. Finally, children have flexibility in the way they choose to describe a picture scene and can select alternate words that are more easily accessible in the target language if there is a specific word that they do not know. Thus, even for children with low language, the social and linguistic features of a discourse task may reduce the challenges associated with maintaining language control in a dual-language context.

Floor effects are also a possibility. In children, and especially in children with low language, maintaining the target language may be sufficiently difficult, even in a single-language context, that the dual-language context does not add much additional difficulty. This may be especially the case in the child’s non-dominant language. Although the observed outcome (no robust effect of context) is the same, different mechanisms may be responsible for the lack of a context effect for children who produce very few cross-language intrusions in either context vs. for children who produce frequent cross-language intrusions in both contexts. Considering the role of linguistic vs. cognitive predictors of language control, it is possible that linguistic skills contribute to maintaining a particular target language in any context, while the ability to shift from one language to another in a dual-language context relates more to cognitive control.

### The Effect of Cognitive Control on Language Control

In the current study, there was no main effect of cognitive control on language control, but cognitive control did moderate the effect of context such that children with more difficulty shifting dimensions in the DCCS also exhibited more cross-language intrusions in the dual-language context compared to the single-language context. This finding diverges from our previous work on language control in children at the single-word level (Gross and Kaushanskaya, 2018), where cognitive control predicted cross-language intrusions overall, regardless of context, and where the effect of context was more robust across children. As described above, there are a variety of differences between a decontextualized picture-naming paradigm and a discourse-level paradigm that may help to explain these discrepant findings. In particular, the presence of only brief auditory cues (“Say” vs. “Diga”) to determine the target language and the need to produce a specific label may increase demands on cognitive control even in a single-language context, yielding a broader role for cognitive control than observed in the current study.

The interaction between context and cognitive control in the current study helps to illuminate what initially appeared to be an absent effect of context. The Adaptive Control Hypothesis (Green and Abutalebi, 2013) describes the dual-language context as the most taxing on language control because it engages additional control processes (salient cue detection, selective response inhibition, task disengagement, task engagement) beyond those required in a single-language context (goal maintenance and interference control). One possibility suggested by the interaction finding is that the dual-language context in our study does still place additional demands on language control. However, children with good cognitive control may be able to use a variety of social and linguistic cues and priming processes to meet these increased demands. Children with poor cognitive control may have difficulty allocating the attentional resources to benefit from the social and linguistic cues signaling the need for a language switch. In addition, pre-exposure to the target language during the confederate’s turn is brief during the dual-language condition, compared to the accumulated exposure over the whole task in the single-language condition. For children who were successful at shifting dimensions on the DCCS, this brief opportunity to listen to the target language may have been sufficient to facilitate the language switch. Children who had difficulty shifting dimensions on the DCCS may not have benefitted from this brief priming effect.

For children with better cognitive control, the increased language control demands in a dual-language context may have benefits for successful language control, even if it is more effortful. Declerck et al. (2017) found that, although bilinguals were more likely to produce cross-language intrusions when they had to switch into a different language to produce a sentence, they were also more likely to go back and correct these cross-language intrusions with the word in the appropriate language. Cross-language intrusions made during non-switch trials, although less frequent, were more likely to be left uncorrected. The authors suggested that monitoring of cross-language intrusions was better when bilinguals were actively switching languages precisely because there was heightened conflict between languages.

### Inter-related Effects of Cognitive Control and Language Ability on Cognitive Control

Our hypothesis that cognitive and linguistic factors may interact in their effects on language control was not supported by the findings of the current study. The effects of cognitive control (overall or in moderating the effect of context) did not differ significantly based on children’s level of language ability. In formulating our hypotheses, we had suggested two possible reasons for a decreased role for cognitive control at lower levels of language ability.

One possibility was that low language ability may be associated with cognitive control difficulties, such that the effects of language ability and cognitive control would be more intertwined at low levels of language ability. In the current study, low language and difficulties with cognitive control did not necessarily go hand in hand. Children who failed the DCCS (*M* = 101.18, *SD* = 14.21) did not exhibit significantly lower language skills than children who passed the DCCS [*M* = 102.80, *SD* = 12.67, *t*(60) = 0.46, *p* = 0.65]. While the number of children with language skills in the lower third who failed the DCCS (10 out of 21) was proportionally greater than for children with mid-level (6 out of 22) and high-level language (6 out of 20), there were still several children with low language who passed the DCCS. Even in the literature on cognitive control in children with language impairment, deficits in shifting skills have been inconsistent (e.g., Dibbets et al., 2006; Im-Bolter et al., 2006; Laloi, 2015; Pauls and Archibald, 2016). In addition, there has been limited work on cognitive control in bilingual children with DLD, and it is possible that they may not show the same level of difficulty with cognitive control as has been observed in monolingual children with DLD (e.g., Peets and Bialystok, 2010).

The other possibility was that limited linguistic skills may sufficiently constrain children’s ability to exercise language control such that any variability in cognitive control would not exert additional effects. Although our analyses do not support this interpretation, a more robust sample of children with low language skills would be necessary to confirm our finding that cognitive control appears to affect language control similarly across the spectrum of language ability. Furthermore, the effect of cognitive control was only observed in moderating the effect of context, compared to the robust overall effect of language ability. Thus, it may be that linguistic skills are the main limiting factor for exercising language control in children at all levels of language ability, not only among children with limited language skills.

## Conclusion and Future Directions

The current study represents an initial step in the attempt to integrate linguistic and cognitive factors in a model of language control in children. We observed an overall effect of language ability on children’s language control and a moderating effect of cognitive control on children’s ability to adjust their language choice to accommodate different monolingual conversation partners in dual-language vs. single-language settings. Taken together, these findings suggest the need for an integrated model of language control in children that incorporates both linguistic and cognitive factors, although linguistic factors may play a more prominent role.

To build on these findings, there are limitations that need to be acknowledged and addressed in future work. First, we only administered one measure of cognitive control, and thus the relationships observed in the current study may be specific to the DCCS and the particular version that we employed. Our chosen outcome measure from the DCCS could also have influenced our findings. To gain a better understanding of the contributions of cognitive control to language control, future work should consider a latent variable approach based on multiple measures and tapping multiple constructs.

Second, we focused our analysis of linguistic predictors of language control on a measure of overall language ability. To better understand why overall language ability had a robust effect on language control, future work should also consider language-specific skills and lexical gaps. Third, there are very likely factors other than language ability and cognitive control that exert an influence on language control. Further work should consider various measures of exposure to each language and to dual-language input as predictors of interest. Social factors are another key area to explore. We suggested that low language ability may affect language control through reduced sociolinguistic awareness, but we did not directly measure pragmatics or social skills in the current study.

Fourth, the relationships observed in the current study were correlational and no claims can be made about directionality or causality. It is possible that children’s experiences in developing language control in different contexts may in turn affect their cognitive control and language skills. Longitudinal work is needed that links changes in linguistic and cognitive skills over time to children’s developing language control skills. The study by Kuzyk et al. (2020) was longitudinal, but floor performance on the cognitive control tasks at the first time point precluded an examination of bi-directional influences of cognitive control and language control on each other over time.

Fifth, the current study included a relatively small number of children at the lower end of the language ability continuum. While the results based on our continuum approach suggest that children with DLD may be at risk for language control difficulties due to their low language skills, we cannot necessarily conclude that children with DLD produce more cross-language intrusions than children with typical development. A larger sample of children with DLD would be necessary to more formally evaluate questions of poorer language control and the role of cognitive control in this clinical group.

Finally, the current study focused on language control in single-language and dual-language contexts with monolingual conversation partners. The findings shed light on contributing factors to language control in these particular contexts but cannot speak to mechanisms of language control in children during conversational code-switching with other bilinguals. Adult models (e.g., Control Process Model of Code-Switching, Green and Wei, 2014; Adaptive Control Hypothesis, Green and Abutalebi, 2013) suggest that dense code-switching contexts may involve a different set of control processes than single-language or dual-language contexts. For a more complete picture of the role of cognitive and linguistic factors in children’s ability to exercise language control in a variety of environments, it will be important to examine questions similar to those addressed in the current study when children interact with other bilinguals in dense code-switching contexts.

## Data Availability Statement

The raw data supporting the conclusions of this article will be made available by the authors, without undue reservation, to any qualified researcher.

## Ethics Statement

The studies involving human participants were reviewed and approved by Education and Social/Behavioral Science IRB, University of Wisconsin-Madison. Written informed consent to participate in this study was provided by the participants’ legal guardian/next of kin.

## Author Contributions

This study was part of the dissertation project conducted by MG under the supervision of MK. MG conceptualized the study and the primary task, conducted the data collection, literature review, and analyses, and drafted the manuscript. MK provided feedback on all stages of the process and assisted in editing the manuscript.

## Conflict of Interest

The authors declare that the research was conducted in the absence of any commercial or financial relationships that could be construed as a potential conflict of interest.

## Footnotes

1 Due to unforeseen scheduling conflicts, the English condition was administered for one child by a different bilingual experimenter who was a non-native speaker of Spanish. For another child, the second half of the Spanish condition was administered by a native Spanish-speaking bilingual experimenter.
