# Supplementary material for: Cognitive and Linguistic Predictors of Language Control in Bilingual Children
Source: Front Psychol. 2020 May 19;11:968. doi: 10.3389/fpsyg.2020.00968 (PMC7248219; doi:10.3389/fpsyg.2020.00968)
Supplement: Supplementary file 1 [file Data_Sheet_1.docx]

**Supplementary Material**

**Stimulus Development for Scripted Confederate Dialogue Task**

The picture scenes in the scripted confederate dialogue task were designed with an animate subject performing an action on an object in a location (or for a recipient) to be described in the form NP VP NP PP (e.g., The girl is hiding the book behind the chair). Nouns and verbs were selected to be early-acquired and high-frequency in both Spanish and English. For the scenes children described, the depicted nouns and verbs had to be produced in both English and Spanish by at least 50% of children between the ages of 24 and 30 months in the American English and Mexican Spanish versions of the MacArthur Communicative Development Inventories, as reported in the online CLEX database (Center for Child Language; Dale & Fenson, 1996; Jackson-Maldonado et al., 2003). For the scenes that children had to match to the confederate’s sentence, the criteria were less strict (produced by at least 50% of children in one language) because no production was required during the task. In addition, scene elements had lemma frequencies of at least 10 tokens per million in the Corpus of Contemporary American English (Davies, 2008) and the Corpus del Español (Davies, 2002).

**Stimulus Norming for Scripted Confederate Dialogue Task:**

The final set of 20 pairs of scenes to be identified and 20 scenes to be described were selected from a larger set of 28 scene pairs and 33 description scenes following an extensive norming process. Twenty-one English speaking adults and six Spanish speaking adults participated in the first phase of the norming process. For the scene pairs, they rated how easy it was to discriminate between the two scenes and how well each scene matched the sentence used to describe it on a 1-5 scale (1 = best match, easiest to discriminate). Prior to norming, the Spanish versions of the sentences had been reviewed by two native speakers of Spanish from Mexico for naturalness and well-formedness. Further modifications to the sentences were suggested by the native speakers who participated in the norming process. For the description component, they rated how easy it was to describe the scene on a 1-5 scale (1 = easy to determine what was happening and think of appropriate words in the relevant language). In addition, they rated all scenes for subjective visual complexity on a 1-5 scale (1 = least complex). Based on this information, the scenes were edited to improve clarity, equalize visual complexity, and make the distinctions between guessing scenes more similar across pairs. In addition, children within and slightly beyond the target age range (ages 4-7) performed the guessing and describing tasks in English (n = 11) and in Spanish (n = 5) on the edited stimuli to identify scene pairs that may be too challenging to discriminate (very slow reaction times, incorrect responses) and description scenes that may be too difficult for young children to describe in English and/or Spanish or that may yield too many different interpretations. A set of 23 scene pairs and 23 description scenes was selected from the larger norming set, and the full paradigm with video confederates was piloted with 4 bilingual children (two 4-year olds, one 5-year old, one 6-year old). Piloting revealed that children from the target age range were able to complete the task.

The final set of 20 scene pairs and 20 description scenes was selected from the reduced pilot set of 23 based on ratings from the norming phase and observations during piloting of children’s ability to discriminate the scene pairs and to describe the description scenes with relatively consistent interpretations. The final set of scene pairs had an average discrimination rating of 1.26 (*SD*=0.14; range 1-1.6) on the 1-5 scale. The match between each scene and its sentence description was rated at 1.04 in English (*SD*=0.06; range 1-1.21) and 1.02 in Spanish (*SD*=0.07; range 1-1.25). The mean difference in visual complexity ratings between members of a pair was 0.04 (*SD*=0.08; range 0-0.25). The description scenes had an average rating of ease of describing of 1.66 (*SD*=0.37; range 1-2.11) in English and 1.44 (*SD*=0.27; range 1-1.88) in Spanish. The description scenes for the stay and switch trials in the dual-language condition did not differ significantly in the ease of description ratings from the norming phase in English (*p* = .97) or in Spanish (*p* = .30). The average visual complexity rating was 2.70 (SD=0.31; range 2.20-3.33).

**Confederate videos**

The confederate videos were recorded by four women from the local community in their late 30s or early 40s (ages 36-41). The two English speakers reported minimal Spanish experience and spoke with a local midwestern American accent. The two Spanish speakers were from Mexico, consistent with the background of most child participants. They reported limited English use (occasionally; a couple hours a day) and limited English proficiency (self-rated 2-4 on a 10-point scale). The confederates were instructed to act as if they were speaking to a child. All guessing sentences were recorded, as well as task instructions, prompts, and feedback. Each confederate was recorded individually in a section of a conference room with a slightly different background (two different bookshelves, white board, presentation screen) so that it would be believable to children that the confederate was in another room but it would not be clear exactly where they were. All guessing trials, prompts, and feedback were spliced from the original recording using Adobe Premiere CC 2014 and organized into the experimental paradigm using E-Prime 2.0 (build 2.0.10.242, Psychology Software Tools, 2012).
