# Supplementary material for: Cognitive and Linguistic Predictors of Language Control in Bilingual Children
Source: Front Psychol. 2020 May 19;11:968. doi: 10.3389/fpsyg.2020.00968 (PMC7248219; doi:10.3389/fpsyg.2020.00968)
Supplement: Supplementary file 2 [file Data_Sheet_2.docx]

*Appendix A*. Elements of description scenes.

| Item | Language | Subject | Action | Object | Location |
| --- | --- | --- | --- | --- | --- |
| 1 | English  Spanish | boy  el niño | wash  lavar | hands  las manos | bathroom  el baño |
| 2 | English  Spanish | girl  la niña | put  poner/guardar | doll  la muñeca | box  la caja |
| 3 | English  Spanish | boy  el niño | eat  comer | bread  el pan | kitchen  la cocina |
| 4 | English  Spanish | man  el señor | wash  lavar | horse  el caballo | tree  el árbol |
| 5 | English  Spanish | boy  el niño | put  poner/guardar | bunny  el conejo | box  la caja |
| 6 | English  Spanish | girl  la niña | sweep  barrer | street  la calle | house  la casa |
| 7 | English  Spanish | lady  la señora | wash  lavar | window  la ventana | bedroom  el cuarto |
| 8 | English  Spanish | man  el señor | buy  comprar | milk  la leche | store  la tienda |
| 9 | English  Spanish | lady  la señora | wash  bañar | dog  el perro | bathroom  el baño |
| 10 | English  Spanish | boy  el niño | eat  comer | cheese  el queso | kitchen  la cocina |
| 11 | English  Spanish | man  el señor | open  abrir | door  la puerta | for lady (recipient)  para la señora |
| 12 | English  Spanish | girl  la niña | put  poner/guardar | glasses  los lentes/anteojos | table  la mesa |
| 13 | English  Spanish | boy  el niño | wash  lavar | cup  la taza | kitchen  la cocina |
| 14 | English  Spanish | girl  la niña | put  ponerse/amarrar | shoe  el zapato | bedroom  el cuarto |
| 15 | English  Spanish | lady  la señora | eat  comer | orange  la naranja | table  la mesa |
| 16 | English  Spanish | girl  la niña | sweep  barrer | floor  el piso | kitchen  la cocina |
| 17 | English  Spanish | lady  la señora | put  poner/guardar | pencil  el lápiz | drawer  el cajón |
| 18 | English  Spanish | man  el señor | close  cerrar | window  la ventana | bathroom  el baño |
| 19 | English  Spanish | boy  el niño | put  ponerse | hat  el sombrero/la gorra | head  la cabeza |
| 20 | English  Spanish | man  el señor | give  dar | present  el regalo | to girl (recipient)  a la niña |
