# Supplementary material for: Cognitive and Linguistic Predictors of Language Control in Bilingual Children
Source: Front Psychol. 2020 May 19;11:968. doi: 10.3389/fpsyg.2020.00968 (PMC7248219; doi:10.3389/fpsyg.2020.00968)
Supplement: Supplementary file 3 [file Data_Sheet_3.docx]

*Appendix B*. Confederate sentences and scenes to be described by participants in the dual-language block (version A) with translations for Spanish sentences provided in italics.

| **Confederate Sentence for Guessing Phase** | **Scene to be Described by Participant** | **Target Language^a^** |
| --- | --- | --- |
| El niño está mirando el oso con su hermana.  [*The boy is looking at the bear with his sister.*] | a lady washing a dog in the bathroom | Spanish |
| La niña está escondiendo el libro detrás de la silla. [*The girl is hiding the book behind the chair.*] | a boy eating bread in the kitchen | Spanish |
| The lady is cooking dinner in the kitchen. | a girl sweeping the street in front of a house | English |
| The man is drinking water in the kitchen. | a boy putting a bunny in a box | English |
| La araña está asustando al niño en el bosque.  [*The spider is scaring the boy in the woods*.] | a man opening a door for a woman | Spanish |
| The dog is looking at the moon through the clouds. | a girl putting her glasses on a table | English |
| The man is looking at the butterfly on the tree. | a lady washing a window in the bedroom | English |
| La señora está trayendo los libros a la escuela.  [*The lady is bringing books to the school*.] | a man buying milk at the store | Spanish |
| El señor está mirando el barco en el agua.  [*The man is watching the boat in the water*.] | a boy putting a hat on his head | Spanish |
| La niña está cocinando pollo en la cocina.  [*The girl is cooking chicken in the kitchen*.] | a man washing a horse by a tree | Spanish |
| The boy is watching the airplane in the sky. | a girl putting a doll in a box | English |
| The man is singing a song at the show. | a boy washing a cup in the kitchen | English |
| The spider is scaring the lady in the living room. | a girl sweeping the floor in the kitchen | English |
| The boy is looking at the sun through the window. | a lady eating an orange at a table | English |
| El niño está cortando las manzanas en la mesa.  [*The boy is cutting apples on the table*.] | a man closing a window in the bathroom | Spanish |
| The lady is looking at the moon in the sky. | a boy eating cheese in the kitchen | English |
| El señor está mirando sus dientes en el espejo. [*The man is looking at his teeth in the mirror*.] | a woman putting a pencil in a drawer | Spanish |
| La niña está leyendo un libro en el sillón.  [*The girl is reading a book on the couch*.] | a boy washing his hands in the bathroom | Spanish |
| The man is pushing the chair into the living room. | a girl putting on her shoe in the bedroom | English |
| La niña está cocinando huevos en la cocina.  [*The girl is making eggs in the kitchen*.] | a man giving a present to a girl | Spanish |

^a^ In version B, each trial occurred in the opposite language.
